# Global health education in Germany: an analysis of current capacity, needs and barriers

**DOI:** 10.1186/s12909-016-0814-y

**Published:** 2016-11-25

**Authors:** Ioannis Kaffes, Fabian Moser, Miriam Pham, Aenne Oetjen, Maya Fehling

**Affiliations:** 1 Cand. med. at Charité – University Medicine Berlin, Charitéplatz 1, 10117 Berlin, Germany; 2 Global Health Education Initiative (GHEI), Berlin, Germany; 3 PhD Cand. at Berlin Graduate School of Social Sciences (BGSS), Humboldt Universität zu Berlin, Luisenstr. 56, D-10117 Berlin, Germany; 4 Médecins sans Frontières Germany, Am Köllnischen Park 1, 10179 Berlin, Germany

**Keywords:** Global health, Education, Medical school, Germany, University

## Abstract

**Background:**

In times of increasing global challenges to health, it is crucial to create a workforce capable of tackling these complex issues. Even though a lack of GHE in Germany is perceived by multiple stakeholders, no systematic analysis of the current landscape exists. The aim of this study is to provide an analysis of the global health education (GHE) capacity in Germany as well as to identify gaps, barriers and future strategies.

**Methods:**

An online search in combination with information provided by student representatives, course coordinators and lecturers was used to create an overview of the current GHE landscape in Germany. Additionally, a semi-structured questionnaire was sent to GHE educators and students engaged in global health (GH) to assess the capacity of German GHE, its barriers and suggested strategies for the future.

**Results:**

A total of 33 GHE activities were identified at 18 German universities. Even though medical schools are the main provider of GHE (42%), out of 38 medical schools, only 13 (34%) offer any kind of GHE. Modules offered for students of other health-related professions constitute 27% of all activities. Most survey respondents (92%, *n* = 48) consider current GHE activities in Germany insufficient. Suggested formats were GHE as part of medical curricula (82%, *n* = 45) and dual degree MD/MPH or PhD programs. Most important barriers mentioned were low priority of GH at faculties and academic management levels (*n* = 41, 75%) as well as lack of necessary institutional structures (*n* = 33, 60%).

**Conclusions:**

Despite some innovative academic approaches, there is clearly a need for more systematic GHE in Germany. GHE educators and students can take an important role advocating for more awareness at university management level and suggesting ways to institutionalize GHE to overcome barriers. This study provides key evidence, relevant perceptions and suggestions to strengthen GHE in Germany.

## Background

Health inequities, effects of climate change on health, the rise in antimicrobial resistance, epidemics such as Ebola [1–3] and other transnational health threats, the multitude of influential global actors beyond traditional bi- and multilateral models as well as the critical role of non-health sectors exceed the capacity of the established discipline of international health. This is due to its focus on tropical medicine, reproductive health, nutrition and hygiene in countries “other than one’s own” [4, 5]. Global Health (GH), on the other hand, is a multidisciplinary field studying and influencing health worldwide through research, practice and policy, including health systems, social, political, environmental and commercial determinants of health, particularly for transnational health matters. It aims to address an unmet need to sustain health in a globalized world where the “distinction between domestic health and foreign health is dissolved” ([6], p. 78).

In order to improve and sustain health locally and globally, a competent clinical and non-clinical GH workforce [6, 7] and, therefore, a well-established global health education (GHE) system is required [8]. In North America and the United Kingdom (UK), there exist a multitude of GHE opportunities, mostly for medical students and medical residents [9, 10], but also for students of other fields [8, 11–14]. Due to the resulting implications for individual well-being, the need for medical schools to include global health issues seems highly relevant, and has been pointed out repeatedly [7, 15]. Even though an agreed-upon definition of GH is missing, there are core competencies of a GHE system described in the literature [8, 16–18]. Arthur et al. [18] suggested topic areas of GH such as global burden of disease, health implications of travel, migration and displacement, social and economic determinants of health, population, resources and environment, globalization of health, healthcare in low-resource settings and human rights in GH.

A study of German medical students by Bozorgmehr et al. [19, 20] identified demand for more GHE among the students as well as knowledge gaps concerning issues relevant to GH such as the Declaration of Alma-Ata, poverty definitions and under-five mortality rates. The German Medical Students’ Association’s “Globalisation and Health Initiative” argued for an integration of GHE into the curricula of medical schools and provided recommendations for the practical implementation of such courses [21]. In 2015, three academies – the German National Academy of Life Sciences Leopoldina, the National Academy of Science and Engineering acatech and the Union of the German Academies of Science and Humanities – issued a joint statement for a comprehensive effort to improve educational and training opportunities in public health and GH in Germany [22]. Despite these perceived gaps and needs, there has not been a comprehensive investigation of the German GHE landscape.

This study aims to provide a first overview of existing educational activities on GH in Germany combined with an analysis of perceived gaps, barriers and future steps. The results should provide guidance for students, academics and educational leaders to better understand and improve GHE in Germany.

## Methods

Two methodological approaches informed by a preceding literature review were used to assess GHE in Germany: an analysis of the current GHE landscape and a survey of stakeholders’ perspectives.

### Landscape analysis

After an initial online scope to identify universities providing GHE, a clear predominance of health-related programs was found. Therefore, the study focused on universities offering degrees in medicine, public health, or health sciences, which were identified through the public university portal Hochschulkompass [23]. This online tool, established by the German Rectors’ Conference, provides a free, comprehensive and up to date catalogue of all degree programs offered at German universities. The identified institutes’ websites were screened for GHE activities using the terms “global health” and the German translation “Globale Gesundheit”. Additionally, the results were verified by searching the same terms combined with the institutes’ names using the search engine Google. This concurrently allowed for the identification and inclusion of activities offered by non-health-related disciplines. The information was cross-checked with responses from student representatives, course coordinators and lecturers. All GHE activities such as seminars, lectures and summer schools that were offered from summer term 2015 onwards and had “global health” or its German translation in their title or official description were included. A comparable methodological approach has been used in a study of this kind before [10]. Seven categories of information were collected: 1) institution, 2) degree program, 3) title, 4) format, 5) timeframe, 6) whether the activity was compulsory (part of the curriculum for all students) or elective (students are required to select among a number of optional courses) or voluntary and 7) institutes or departments that were involved. Both elective and compulsory activities were considered curricular activities.

### Stakeholder analysis

A semi-structured survey with 13 quantitative and six open-ended questions was administered using purposive sampling. Two groups of GHE-relevant stakeholders were chosen for this survey as they were considered informed in GHE and Germany’s university setting:

1. GHE educators: academics involved in GH teaching at German medical, public health and other relevant faculties or institutes identified through the landscape analysis and snowball sampling.
2. GH-engaged students: students enrolled in GH-associated groups listed by the German Medical Students’ Association’s “Globalisation and Health Initiative”.

Because GHE in Germany is partially in English, educators and students might not necessarily be German and are assumed to be fluent in English, the questionnaire was done in English. The questions aimed to gain information related to GHE, in terms of 1) perceptions of the current capacity, 2) possible barriers and 3) suggested future strategies for GHE in Germany. They were informed by a preceding literature review of international GHE, GHE in Germany and German GH activities, tested by individuals akin both target groups and modified upon their comments to the final version. It was sent via email and filled out in PDF format or using SoSci Survey software. All questionnaires were anonymized for analysis.

Quantitative data were analyzed using Microsoft Excel (2011). Likert items were interpreted as ordinal data and qualitative data coded manually. The survey was approved by the ethics committee of Charité – University Medicine Berlin, Germany. Consent was obtained from all participants and no incentives were provided.

## Results

Data were collected from March to September 2015.

### Landscape analysis

The search on hochschulkompass.de identified 38° in medicine, 20° in the health sciences, 15° in public health and two degrees combining public health and the health sciences. These degrees were offered at 58 universities in Germany. Investigation of the degree programs rendered 26 GHE activities, and seven additional GHE activities not directly related to the aforementioned degree programs were found.

All 33 GH-related activities are provided by or in cooperation with 18 German universities. Out of those activities, 14 (42%) are offered as part of medical schools’ curriculum. Semester-long, elective seminar series represent the majority of those curricular activities (*n* = 10, 30%).

The medical faculty of the University of Hamburg offers a six semester-long elective course that integrates GH into a broader curriculum of intercultural competence and international medicine. The final year elective “Tropical Medicine and Global Health” at the University of Würzburg constitutes another opportunity for medical students to incorporate GH into their curriculum by combining clinical and project work. Two medical schools have mandatory GHE offered as a lecture on public health at the University of Bonn that includes GH topics and a seminar series on GH ethics at the University of Erlangen-Nuremberg. The medical faculty of the University of Giessen employs a voluntary GH-focused curriculum (Schwerpunktcurriculum “Global Health”), which is a combination of different teaching formats such as lectures, seminars and studies abroad. The student-led “Globalisation and Health Initiative” (GandHI) at the University of Aachen as well as the University of Heidelberg’s Society Georg Forster for Global Health offer extracurricular GH activities for medical students. Other voluntary extracurricular activities open to students of all disciplines are the “Global Health Student Group”, the “Global Health Summer School”, the “Global Health Conference” in Berlin and the “Summer Academy – Global Health and Tropical Medicine” at the Medical Mission Institute Würzburg. Overall, there are GHE opportunities at 13 of 38 (34%) medical schools in Germany.

In addition to medical schools, six universities with health-related degree programs like public health and health sciences offer GHE activities: five GH-specific modules (15% of total GHE activities) and four modules (12%) partly addressing GH. Furthermore, an elective GH lecture series and an elective seminar series for Master of Arts and PhD programs, respectively, are offered by the Department of Development Economics at the University of Göttingen.

The University of Freiburg will offer the first interdisciplinary Master of Science program in GH starting in October 2016.

Overall, some universities offered detailed online course descriptions, whereas others lacked accessible and updated information, requiring further investigation through direct contact.

A summary of all identified GHE activities can be found in Table 1.

**Table 1:** Overview of global health education activities in Germany

University	Degree program	Title	Format	Time	Modality (Compulsory/ Elective/Voluntary)	Institutes and/or Departments
Charité – University Medicine Berlin	Medicine	Global Health Basics - Medical activities in times of globalization I^a^	Seminar	16 x 90 min	Elective	Institute for Social Medicine, Epidemiology and Health Economics
	Medicine	Global Health Advanced - Medical activities in times of globalization II^a^	Seminar	4 x 90 min	Elective	Institute for Social Medicine, Epidemiology and Health Economics
	Master of Public Health	Global Health Databases^b^	Part of the Module “Advanced Public Health research methods”^a^	70 h	Elective	Berlin School of Public Health
	N/A	Global Health Student Group	Student Group	2–4 sessions per month	Voluntary	Organized by students from different disciplines
	N/A	Global Health Summer School	Summer School	7 days	Voluntary	Institute for Social Medicine, Epidemiology and Health Economics, IPPNW^c^
	N/A	Global Health Conference	Conference	1 day	Voluntary	Institute for Social Medicine, Epidemiology and Health Economics, IPPNW^c^
Hamburg University of Applied Sciences	Master of Public Health	Global, European and German Public Health	Module	180 h	Compulsory	Faculty of Life Sciences, Department of Health Sciences
	Master of Health Sciences	Health Economics and Global Health	Module	180 h	Elective	Faculty of Life Sciences, Department of Health Sciences
Heidelberg University	Medicine	Global Health	Seminar	2 weekends	Elective	Institute of Public Health
	Medicine	Society Georg Forster (Society for Global Health)^a^	Seminar	At least 4 sessions per term	Voluntary	Interdisciplinary
Ludwig-Maximilians University Munich	Medicine	Global Health	Seminar and exercises (e.g. microscope)	10 x 90 min	Elective	Department of Infectious Diseases and Tropical Medicine
	Master of Public Health; Master of Science Epidemiology; PhD International Health	Global Public Health	Module (2 courses: Global Public Health situation^a^, Public Health policies in a globalized world^a^ )	180 h	Elective	Institute of Medical Data Processing, Biometrics and Epidemiology, international guest lecturers
RWTH Aachen	Medicine	Globalisation and Health Initiative (GandHI)	Student group, project team of the German Medical Students’ Association’s Public Health Working Group (Summer School, Basics Seminars)	3 seminars per term, 1 summer school per term	Voluntary	Organized by Medical Students
Technical University Munich	Bachelor of Science Health Science	Global Health	Module (3 seminars)	180 h	Elective	Chair of Sociology of Diversity, Faculty of Sport and Health Sciences
University of Applied Sciences, Fulda	Master of Science Public Health; Master of Science Public Health Nutrition	Globalization and Health^a^	Module	300 h	Elective (M.Sc. Public Health), Compulsory (M.Sc. Public Health Nutrition)	Department of Nursing and Health Sciences
University of Bonn	Medicine	Global Health	Seminar	16 x 180 min	Elective	Institute of Medical History
	Medicine	Global Health: From Colonial Medicine to Primary Health Care and the Millennium Development Goals (part of a lecture on Public Health)	Lecture	60 min	Compulsory	Institute of Medical History
University of Bremen	Master of Public Health – Health Promotion and Prevention; Master of Science Community and Health Nursing	Global Health^b^	Part of the Module “Public Health – Advanced 2”^a^ (lectures)	14 x 90 min	Elective	Department of Human and Health Sciences
	Master of Public Health – Health Care, Economics and Management; Master of Science Epidemiology	Ethics in Global Health Politics^b^	Part of the Module “Public Health – Advanced 2”^a^ (lectures)	14 x 90 min	Elective	Department of Human and Health Sciences
	Bachelor of Public Health/Health Sciences	“ I want to work in development aid” – on Global Health and development cooperation^a^	Part of the module “Target group – specific Prevention and Health Promotion”^a^ (Seminars, lectures)	14 x 90 min	Elective	Department of Human and Health Sciences
University of Erlangen-Nuremberg	Medicine	Global Health Ethics. Theory and Practice of International Health Care^a^	Seminar (+ research colloquium)	Twice every term (10,5 h)	Compulsory (Colloquium Voluntary)	Institute for History of Medicine and Medical Ethics
University of Freiburg	Medicine	Global Health	Seminar	30 h	Elective	Department of Psychosomatic Medicine and Psychotherapy, Institute for Environmental Medicine and Hospital Hygiene
	Master of Science in Global Urban Health (planned for Oct 2016)	Master of Science in Global Urban Health	Master of Science	1 year	Voluntary	Interdisciplinary^d^
University of Giessen	Medicine	Global Health-focused curriculum^a^	Focused curriculum (combination of clinical electives, at least 4 advanced seminars, compulsory study time abroad, theme nights, lecture events, conference visits)	4–6 clinical terms	Voluntary, can partly be recognized as elective course	Institute of the History of Medicine; Institute of Hygiene and Environmental Medicine; Chair for Nutrition in Developing Countries of Faculty of Agricultural Sciences, Nutritional Sciences and Environmental Management
University of Göttingen	Master of Arts programs (economics and business oriented)	Global Health	Lectures (+ exercises)	12 lectures (42 h)	Elective	Development Economics
	Ph.D. Programs (economics)	Advanced Global Health	Seminar	8 x 4 h	Elective	Development Economics
University of Greifswald	Medicine	Global Health and Tropical Medicine^a^	Seminar (including opportunity for an international elective)	10 seminars (39 h)	Elective	Institute for Community Medicine – Section Family Medicine
University of Hamburg	Medicine	International Medicine/Global Health as part of the Second Track “Intercultural competence and International Medicine – intermed”^a^	Second track (series of complementary electives aimed at fostering students’ insight into the scientific aspects of medicine based on their personal preferences)	5th–10th term, 2 weeks per term	Elective	Institute of General Practice/Primary Care, Department of Medical Sociology
University of Marburg	Medicine	Global Health	Seminar	42 h	Elective	Department of Primary Care, Preventive and Rehabilitation Medicine
University of Münster	Medicine	Global Child Health	Seminar	14 x 90 min	Elective	Department for Pediatric Hematology and Oncology
University of Würzburg	Medicine	Global Health^a^	Seminar	6 x 3 h	Elective	Department of Tropical Medicine of Medical Mission Hospital in Würzburg, Medical Mission Institute
	Medicine	Tropical Medicine and Global Health^a^	Final year elective (combination of clinical work, social commitment and project work)	3,5 months	Elective	Department of Tropical Medicine of Medical Mission Hospital in Würzburg, Medical Mission Institute
	N/A	Summer academy – Global Health and Tropical Medicine^a^	Summer academy (lectures, group work, simulation games, exercises)	12 x 8 h	Voluntary	Medical Mission Institute Würzburg

^a^Original title in German

^b^No response received through cross-check

^c^International Physicians for the Prevention of Nuclear War

^d^Lecturers from a variety of disciplines/ organizations: Department of History, East Asian History; Institute for Cultural Anthropology/ Folkloristics; Institute for Ethnology; Oriental Seminar; Anthropology; Department of Psychosomatic Medicine and Psychotherapy; Department of Environmental Health Sciences; Department of Biological Anthropology; Department of Medical Microbiology and Hygiene; Center for Infectious Diseases; Department of Medical Biometry and Statistics; Department of Palliative Care; German Cochrane Center; Department of Medical Psychology and Medical Sociology; Institute of Environmental Social Sciences and Geography; Institute of Sports and Exercise Science; Chair of Meteorology and Climatology; Department of Marketing and Health Care Management; Migration research; Department of Urban and Regional Planning; WHO; German Society for International Cooperation (GIZ); NGOs et al

### Stakeholder analysis

Thirty-four GH educators were identified from 20 German universities. All received the questionnaire and 27 GH educators responded (79%) from 18 different universities. In addition, the questionnaire was sent to 38 students from GH-associated initiatives with a response rate of 74% (*n* = 28) from 14 universities. From the 55 received questionnaires, results of seven of the survey questions were analyzed because they were considered most relevant to the research question of this publication. Percentages of results were adjusted according to the number of respondents for single answers.

#### Academic background of study participants

Most educators had degrees in human medicine (*n* = 25, 93 %), public health (*n* = 13, 48%) and advanced training in tropical medicine (*n* = 4, 15%). Further, international health, economics, ethics, epidemiology and political sciences (*n* = 2, 7% respectively) as well as biology, theology and psychology (*n* = 1, 4% respectively) were named. Educators held a range of academic positions including project and teaching coordinators, guest lecturers, junior, assistant and university professors, senior lecturer and heads of department.

Students’ background was primarily human medicine (*n* = 24, 85%), two students studied pharmaceutical sciences (7%) and two students did not specify their programs. For more detailed survey participant characteristics see also Table 2.

**Table 2:** Characteristics of survey participants (entry numbers are indicated in parentheses; multiple answers were possible)

Educator characteristics	
Average age	49
Affiliated Institutions	- Akkon University for Human Sciences - Berlin School of Public Health - Charité – University Medicine Berlin - German Leprosy and Tuberculosis Relief Association (DAHW) - Hamburg University of Applied Sciences - Leibniz Institute for Prevention Research and Epidemiology - Ludwig-Maximilians-University Munich - Medical Mission Institute Würzburg (2) - Ulm University - University of Applied Sciences Fulda - University of Bonn - University of Bremen (2) - University of Erlangen-Nuremberg - University of Freiburg (3) - University of Giessen - University of Göttingen - University of Greifswald - University of Heidelberg (2) - University of Marburg (2) - University of Münster - World Health Organization
Affiliated Departments (four educators did not specify their department)	- Biological Anthropology - Center for Medicine and Society - Department of General Practice - Department of Psychosomatic Medicine and Psychotherapy - Economics (2) - Epidemiology and Health - Ethics and History of Medicine (3) - Family Medicine - Health Economics - Health Sciences - Health Systems and Public Health - History of Medicine - Humanitarian Assistance - Institute for Community Medicine - Institute for Social Medicine - Institute of Medical Informatics, Biometry and Epidemiology - International Disaster and Catastrophe Relief - Nursing and Health - Pediatrics (2) - Prevention and Evaluation - Public Health - Tropical Medicine - Tropical Medicine and Global Health Teaching Unit
Degrees	- Human Medicine (25) - Public Health (13) - Advanced training in tropical medicine (4) - International Health (2) - Economics (2) - Ethics (2) - Epidemiology (2) - Political sciences (2) - Biology (1) - Theology (1) - Psychology (1)
Positions (two educators did not specify their position)	- Head of department (4) - Senior Lecturer (2) - Guest Lecturer (3) - Professor (7) - Assistant Professor (2) - Junior Professor (1) - Programme Coordinator (5) - Others (4)
Student characteristics	
Average age	26
Universities (one student did not specify the university)	- Charité – University Medicine Berlin (3) - University of Bonn - University of Freiburg (3) - University of Giessen (3) - University of Göttingen - University of Heidelberg (3) - University of Jena - University of Mainz (2) - University of Marburg - University of Regensburg (3) - University of Tübingen (2) - University of Würzburg (2) - Ruhr University Bochum - Technical University Munich
Degree program (two students did not specify their degree program)	- Human Medicine (24) - Pharmacy (2)

#### Germany’s GHE performance poor compared to the UK and Sweden

Compared to the UK, most educators (*n* = 22, 88%) and students (*n* = 13, 76%) think of Germany’s GHE performance to be poor or very poor. Compared to Sweden, 13 educators (52%) and 9 students (67%) consider German GHE poor or very poor.

#### Insufficient GHE at institutions and Germany overall

**Fig. 1:**
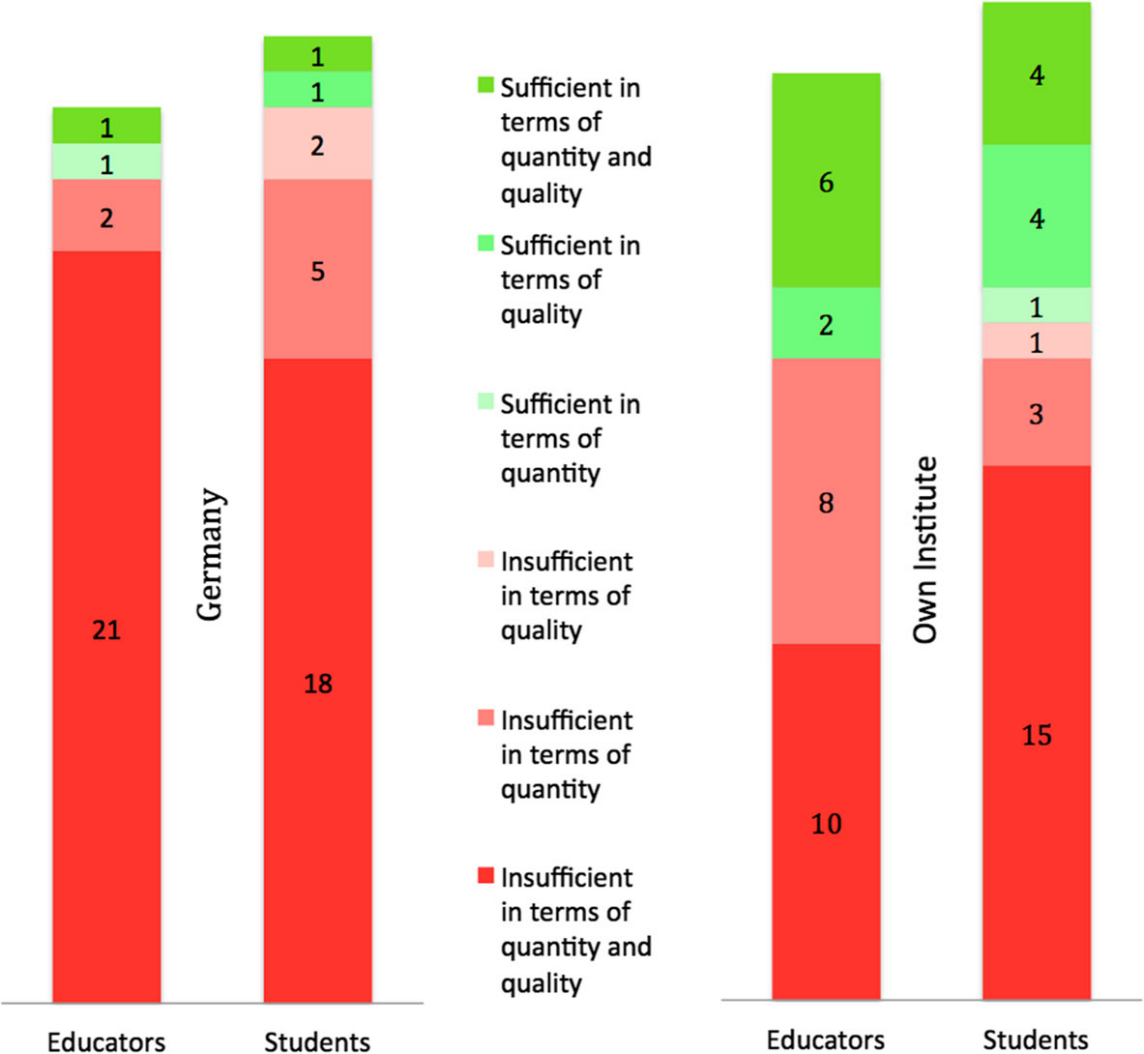
Sufficient GHE at your institution and in Germany? Perception of the sufficiency of GHE in Germany and at the respondent’s own institution (number of respondents)

Current GHE opportunities in Germany were perceived insufficient in quality as well as quantity by 84% (*n* = 21) of educators and 67% (*n* = 18) of students (Fig. 1). At their own institutes, around two-thirds of educators (*n* = 18, 69%) and students (*n* = 18, 68%) considered existing GHE programs insufficient.

#### Reasons for more GHE in Germany

**Fig. 2:**
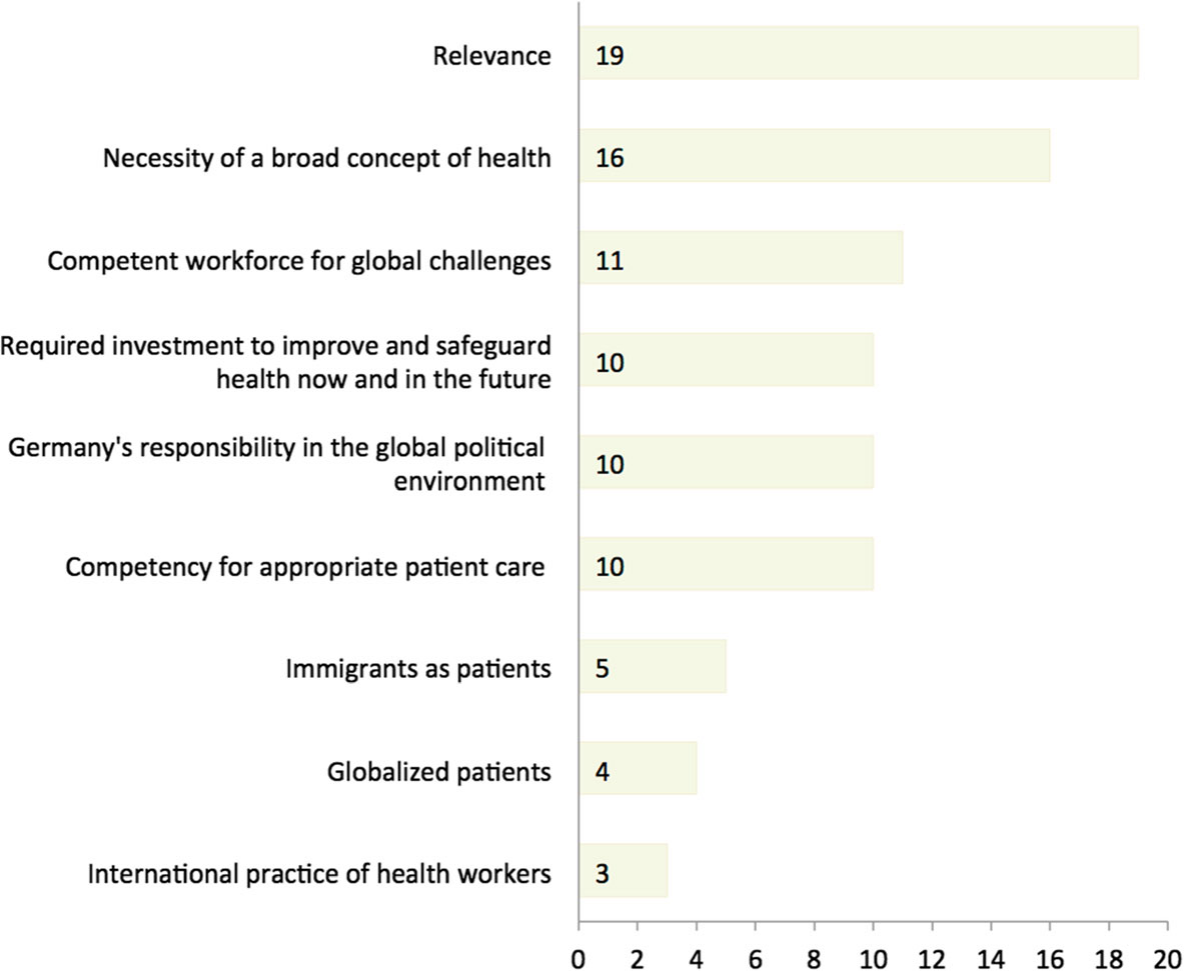
Selected reasons for more GHE, by number of respondents

The survey participants gave multiple reasons why more GHE should be provided (Fig. 2). The most common reason stated was the general relevance of GH (*n* = 19, 35%). Second most frequently reported reason to increase GHE (*n* = 16, 29%) was the necessity for a broader concept of health, including social determinants of health, a population approach and the opportunity of “re-socializing” medical education. Respondents also indicated there is a need for a competent workforce for increasing global challenges such as antimicrobial resistance, health disparities and climate change (*n* = 11, 20%); GHE as required investment to improve and safeguard health now and in the future (*n* = 10, 18%); and Germany’s responsibility in the global political environment (*n* = 10, 18%).

#### More GHE is planned at institutions

From 14 different universities, 82% (*n* = 19) of educators reported plans to expand GHE. Overall, plans for more future inter-institutional cooperation were mentioned by educators from five different universities including cooperation with other faculties for interdisciplinary seminars, between universities as well as with international organizations and scientific institutes. Respondents from four universities reported plans for additional GH Master degrees – two for medical students only, one of which planned as a dual Medical Doctor (MD)/Master of Science (MSc) degree. Furthermore, four universities and several student initiatives intend to increase the number of GHE activities.

#### Preferred curriculum format for GHE

More than 75% (*n* = 22) of students and 85% (*n* = 23) of educators suggested that GHE should be both part of the core curricula of medical schools as well as offered in different GH study formats such as dual degrees (MD/Master of Public Health (MPH) or MD/MSc) (*n* = 27, 56%), MPH and PhD programs (both equally *n* = 23, 48%) and short GH courses (*n* = 17, 40%). Joint degrees with other disciplines such as social science, cultural sciences and law were also mentioned. Three students recommended integrating GHE into the core curricula of pharmaceutical schools and other health sciences.

#### Public funding preferred for financing GHE

Public funding without tuition fees was most often selected by 51% students (*n* = 14) and 56% educators (*n* = 14) as preferred financing mechanism for GHE in Germany. Seven students and six educators (26 and 24% respectively) preferred GHE with tuition fees for private and public institutions. Six students (22%) and none of the educators favored public-private partnerships as a financing method.

According to several individuals, financial support from the private and public sector as well as institutions and organizations should be granted as future employers benefit from a qualified GH workforce.

#### Barriers to GHE in Germany and how to address them

The most commonly identified barrier to GHE in Germany was the low priority given to GH by faculty members and academic management levels (students *n* = 23, 82%; educators *n* = 18; 67%). The second most selected impediment reported by students and educators alike was the lack of institutional structures for GHE (*n* = 17; 60% and *n* = 16; 59% respectively). Individual comments underscored the lack of institutional support for additional GHE at universities, including administrative difficulties, particularly in accreditation of new programs, and that German GHE “depends very much on local initiatives” and “on a few motivated people”. Lack of GH experts teaching at universities was noted by 54% of students (*n* = 15) and 33% of educators (*n* = 9); 43% of students (*n* = 12) and 30% of educators (*n* = 8) noted a general lack of GH experts in Germany. One educator added, “There are hardly any GH research groups in Germany”. The lack of an interdisciplinary approach in GHE seems to be a more important obstacle than the lack of finance, as numerous educators reported (*n* = 14; 52% versus *n* = 11; 41% respectively). As suggested by one educator, an interdisciplinary center “at faculty, university or regional level” could prevent GH from being “a dead discipline of its own”.

Others commented that in Germany “health is seen as the responsibility of the medical field” and “even public health does not play a major role”.

Additional barriers to considering GH or GHE as a professional option were the uncertainty of career opportunities in GH as well as the lack of academic career opportunities for those working internationally in GH.

Proposed steps by students and educators to overcome these barriers are listed in Table 2. Overall an increased priority of GH and GHE at all decision-making levels seemed the most important way towards better GHE in Germany. Hereby, advocacy at universities, federal and state bodies were most often chosen by 15 respondents (28%). To improve GHE, more federal and state funding was marked as necessary by 11% (*n* = 6). More cooperation and collaboration with other academia and organizations, clear career perspectives for graduates as well as for GH workers interested in teaching were chosen solutions to further enhance German GHE (Table 3).

**Table 3:** Proposed measures to overcome existing barriers, as given by respondents (% of all respondents)

Priority setting of GH and GHE at decision-making level
□ Advocacy at universities, federal and state bodies (e.g. ministries of education) (*n* = 15, 28%) □ Students as active parts in the advocacy work for more GHE (*n* = 3, 5%) □ Increasing public awareness of GH issues (*n* = 2, 4%) □ Consensus on a definition of GH (*n* = 1, 2%)
Funding
□ Increased funding for current GH courses by federal and state level (*n* = 6, 11%), □ Incentives for universities to invest in GHE through competitive funding mechanisms (*n* = 2, 4%) □ Investment into new curricula at medical schools including GHE (*n* = 1, 2%)
Stronger cooperation and collaboration in the provision of GHE
□ Stronger cross-faculty collaboration for interdisciplinary GHE (5%, *n* = 3) □ More collaboration between GH(E) experts in Germany (4%, *n* = 2) □ More collaboration of academia with (international) organizations/scientific institutes operating in the field of GH (e.g. NGOs, bi- and multilateral agencies to ensure professional expertise in GHE programs) (2%, *n* = 1)
Career prospects
□ Create new university career options for internationally practicing professionals who have not necessarily pursued an academic career before (5%, *n* = 3) □ Transparency on career options for GH graduates (e.g. career fairs, academia establishes contacts with international organizations and institutions) (4%, *n* = 2)

## Discussion

This analysis paints a rather sobering picture of the current landscape, yet provides reason for optimism regarding future opportunities for GHE in Germany.

Despite recent efforts to improve GHE, the study suggests that these are still insufficient. With only one-third of medical schools and less than a third of all health-related degree programs in Germany offering some kind of education in GH, there is clearly room for improvement. While the German political commitment to GH is increasing [24, 25], GHE as an investment to safeguard health at home and abroad seems not enough of a priority, as confirmed by the vast majority (92%) of academic GH educators and students participating in our study. Countries in North America and the UK particularly, outstrip Germany’s GHE activities in the number and degree options as well as research on GHE [9]. Only a few German universities invest in any GHE activities, and the modalities of the existing formats show high variations between institutions. Whereas numerous extracurricular activities underscore a general interest in GH, the high percentage of elective courses combined with the scarcity of mandatory courses are indicative of a lack of institutional emphasis and prioritization of GH. Integrating GH into the core curricula of medical schools – as suggested by the majority of survey participants – could help standardize the format and content of GHE and, hence, limit variations that might impact efficiencies and possibly quality of education. The importance of GHE in core curricula of medical schools has been highlighted by Houpt et al. [26], who suggested core competencies relevant to all medical students, regardless of their career objectives. However, in light of the fact that there is currently only one mandatory seminar and lecture found in this study, implementation of GHE into the core curricula of all medical schools appears ambitious. Prioritizing certain GH topics and competencies, which was an essential step towards advancing GHE in the United States (US), Canada and the UK [27, 28], could be one way for Germany as well. Medical schools could also benefit from an international dialogue with universities, academic networks such as the Consortium of Universities for Global Health and other working groups in order to establish additional GHE opportunities. Strong networks and innovative teaching methods like e-learning are recognized opportunities to increase GHE despite limited teaching capacity [29, 30].

Various educational formats within medical schools could help students with aspirations to pursue a career in GH service, program delivery, research and policy [31]. One example and possible model for others is the GH-focused curriculum at the University of Giessen, which could pave the way for dual degrees.

Dual degrees such as MD/MPH or MD/MSc degrees were the most preferred formats identified in this survey. These degrees could offer another opportunity to prepare medical students for taking a population-based approach to health, to navigate complex political and socioeconomic environments and gain further skills in research and implementation [32]. Currently, no German medical school offers dual degrees, in contrast to the US, where dual degrees are available at more than 80 universities [33].

Our results make a bold call for greater GHE within medical schools, but they equally express the need for more specialized education and postgraduate degrees beyond medical school. In countries like the UK and the US, these degrees are well-established [10, 34], whereas the German landscape is far more scattered and less coherent. The survey respondents, who bemoaned the limitations of German GHE compared to other countries, confirmed this gap.

Medical school remains the most important stakeholder with regard to GHE in Germany, or, as described by one respondent, health is generally seen as “the responsibility of the medical field”. Whereas medical schools and clinicians certainly play an important role in all health aspects, GH requires a more holistic view to understanding and addressing GH challenges. An interdisciplinary approach is essential for GH [35] and has been mostly neglected by German universities. The elective lecture and seminar series at the Department of Development Economics at the University of Göttingen gives reason for optimism by indicating interest and expertise outside the health professions with the potential for cross-disciplinary collaboration. In this vein, the Master of Global Urban Health at the University of Freiburg incorporates multiple disciplines and represents an example of a more systematic and comprehensive curriculum for GH. The complexity of GH certainly requires a multi-layered GHE system that increases general awareness of this important topic and, at the same time, provides opportunities to choose a GH career path consistent with a student’s background and aspirations.

Our analysis shows that GHE in Germany is hindered by a multitude of barriers such as a low priority of GH at faculty and academic management levels, lack of institutional structures, lack of an interdisciplinary approach and a shortage of GH educators. Whereas appropriate funding is clearly necessary, this study underscores that conceptual and structural issues are perceived as even higher obstacles.

Low priority at faculty and academic management levels could be explained by many factors. GH educators and student responses suggest that there is not enough awareness of the different determinants of health and the relevance of public health. Since public health is an essential element of GH [5], this lack of awareness translates into an equally low or even lower priority for GHE.

Institutional difficulties perceived in this study, such as a lack of a coherent understanding of GH and a lack of institutional support for cross-disciplinary collaboration, have been described in academic GH structures elsewhere as, for example, at the University of Toronto [36]. University-wide GH centers, which are established at various North American universities, could be an organizational form to overcome these difficulties and “have expanded the disciplinary framework (for GH) beyond the health professions,” as noted by Merson and Page ([34], p. 2). Similar university-wide GH structures also seemed to be most preferred in this study. In fact, in a joint statement, three different academies (2015) made specific structural suggestions to improve coordination and collaboration among existing institutions involved in public and GH research, education and practice. The suggestions ranged from a rather loose “Public and Global Health Network Germany” between universities to a “German Centre (or Foundation) for Public and Global Health” to coordinate a network of affiliated institutions [22].

A general lack of German GH experts and those involved in teaching and research were other noted barriers. The survey suggested that universities fail to recruit internationally experienced GH workers as GHE educators. Respondents recommended creating new university-level career options for internationally practicing professionals who are not necessarily pursuing an academic career and direct collaboration with GH organizations, institutes and agencies. An inter-institutional collaboration might increase teaching capacity with relevant professional experience while at the same time helping to bridge GHE to research and practice. Two universities plan to collaborate with the German Agency for International Development and the German Centre for Development – an opportunity that could provide students insights into possible career options and address the needs of future employers.

As health education is globally challenged by increasing and complex demands in the 21st century, Germany is one of many countries yet to find a thorough academic response. Barriers described could hamper progress towards improvements in medical education and an effective GHE system in a similar manner elsewhere. In spite of the prerequisite to set local priorities and ensure diversity in educational systems for health [7], ideas and strategies for improvement outlined throughout this study could be of interest to other countries within the European Union and beyond.

### Limitations

There are four main limitations to this study. First, the number of participating students was small compared to the overall amount of medical students and actual numbers of GHE participants were not available, due to the lack of consistent and reliable information, which hinders a definitive assessment of whether sample size was enough for this group. However, considering the alignment of answers despite the different institutes these participants were coming from, we assume that the results provide a valid picture of the current perception. Second, the majority of educators and all students surveyed for this study had health-related backgrounds and all participants formed an active part of the German GHE landscape, limiting the perspective of the survey results. This shortcoming notwithstanding, the issues addressed in the questionnaire required a profound insight into GHE in Germany, rendering the selected participants crucial to furthering a constructive dialogue on the issue at hand. Third, the landscape analysis did not identify courses on international health, even though GH topics overlap with the discipline of international health, which focuses on tropical medicine, reproductive health, nutrition and hygiene in countries “other than one’s own” [5]. The distinction was nonetheless considered necessary in order to fulfill the aim of this study, namely, to create an overview of the field of GH in Germany that extends the disciplinary range and focus to transnational health aspects, emphasizes global cooperation and aims for health equity among all nations and people [5]. Fourth, the survey did not provide a definition of GH, which might have caused some inconsistency in what participants considered as “global health” and which activities were included in the landscape analysis. However, given the general lack of a unanimously agreed definition and the incoherence of the academic content of GHE, the chosen approach was deemed the most conducive to achieving a broad reach without causing a dilution of the topic.

## Conclusion

There is clearly a need for more systematic GHE in Germany, which, at the moment, is impeded mainly by a lack of institutional priority and structure. With increasing relevance of GH also in high-income countries, GH educators and students represent one of the most important advocates for GHE at all political levels. Together with decision-making stakeholders, they should engage in a debate on GH curricula with a focus on core competencies, an interdisciplinary approach and best teaching formats. For key stakeholders, this overview of GHE in Germany and understanding of the perceptions of students and educators may serve to sway decision-makers and institutionalize the subject. Additionally, it helps those interested in GHE, whether as students or educators, to make career choices. In spite of the identified concerns about GHE in Germany, this study also provides positive examples throughout the academic landscape that are encouraging and can serve as models for future efforts.

Clearly, this work can only be a first step towards a systematic strengthening of GHE in Germany, which has to be followed by future research, the exchange of knowledge and action, building on the information and insights gained through this study. Besides innovative curricula and teaching formats, research efforts should focus on the evaluation of GHE programs, processes to overcome barriers identified in this study and particularly models enabling conducive interdisciplinary and interinstitutional collaboration.

## Abbreviations

GH: Global health
GHE: Global health education
IPPNW: International Physicians for the Prevention of Nuclear War
MD: Doctor of Medicine
MPH: Master of Public Health
MSc: Master of Science
PhD: Doctor of Philosophy
RWTH Aachen: Rheinisch-Westfälische Technische Hochschule Aachen
UK: United Kingdom
US: United States

## References

[1] CSDH (2008). Closing the gap in a generation: health equity through action on the social determinants of health. Final Report of the Commission on Social Determinants of Health.

[2] Watts N, Adger WN, Agnolucci P, Blackstock J, Byass P, Cai W (2015). Health and climate change: policy responses to protect public health. Lancet.

[3] WHO (2014). Antimicrobial resistance: global report on surveillance 2014.

[4] Brown TM, Cueto M, Fee E (2006). The World Health Organization and the transition from “International” to “Global” Public Health. Am J Public Health.

[5] Koplan JP, Bond TC, Merson MH, Reddy KS, Rodriguez MH, Sewankambo NK (2009). Towards a common definition of global health. Lancet.

[6] Fineberg HV, Hunter DJ (2013). A global view of health – An unfolding series. N Engl J Med.

[7] Frenk J, Chen L, Bhutta ZA, Cohen J, Crisp N, Evans T (2010). Health professionals for a new century: transforming education to strengthen health systems in an interdependent world. Lancet.

[8] Jogerst K, Callender B, Adams V, Evert J, Fields E, Hall T (2015). Identifying interprofessional global health competencies for 21st-century health professionals. Ann Glob Health.

[9] Liu Y, Zhang Y, Liu Z, Wang J (2015). Gaps in studies of global health education: an empirical literature review. Glob Health Action.

[10] Harmer A, Lee K, Petty N (2015). Global health education in the United Kingdom: a review of university undergraduate and postgraduate programmes and courses. Public Health.

[11] Conrad PA, Mazet JA, Clifford D, Scott C, Wilkes M (2009). Evolution of a transdisciplinary “One Medicine–One Health” approach to global health education at the University of California, Davis. Prev Vet Med.

[12] Peluso MJ, Hafler JP, Sipsma H, Cherlin E (2014). Global health education programming as a model for inter-institutional collaboration in interprofessional health education. J Interprof Care.

[13] Palmer VS, Mazumder R, Spencer PS (2014). Interprofessional Global Health Education at Oregon Health and Science University: The Interprofessional Community Health and Education Exchange (iCHEE) Experience. Acad Med.

[14] Villafuerte-Galvez J, Curioso WH (2007). Teaching global health at the frontlines. PLoS Med.

[15] Margolis CZ, Deckelbaum RJ, Henkin Y, Alkan M (2002). Bringing global issues to medical teaching. Lancet.

[16] Brown LD (2014). Towards defining interprofessional competencies for global health education: drawing on educational frameworks and the experience of the UW-Madison Global Health Institute. J Law Med Ethics.

[17] Ablah E, Biberman DA, Weist EM, Buekens P, Bentley ME, Burke D (2014). Improving Global Health Education: development of a global health competency model. Am J Trop Med Hyg.

[18] Arthur MAM, Battat R, Brewer TF (2011). Teaching the basics: core competencies in global health. Infect Dis Clin North Am.

[19] Bozorgmehr K, Schubert K, Menzel-Severing J, Tinnemann P (2010). Global Health Education: a cross-sectional study among German medical students to identify needs, deficits and potential benefits (Part 1 of 2: Mobility patterns & educational needs and demands). BMC Med Educ.

[20] Bozorgmehr K, Menzel-Severing J, Schubert K, Tinnemann P (2010). Global Health Education: a cross-sectional study among German medical students to identify needs, deficits and potential benefits (Part 2 of 2: Knowledge gaps and potential benefits). BMC Med Educ.

[21] bvmd, Globalisation and Health Initiative (GandHI) (2009). Lehre am Puls der Zeit – Global Health in der medizinischen Ausbildung: Positionen, Lernziele und methodische Empfehlungen.

[22] German National Academy of Sciences Leopoldina, acatech, National Academy of Science and Engineering and Union of the German Academies of Sciences and Humanities (2015). Public Health in Germany – Structures, Developments and Global Challenges. Halle (Saale).

[23] Hochschulkompass. http://www.hochschulkompass.de/. Accessed 15 May 2015.

[24] Kickbusch I (2015). What explains Germany’s new role in global health?. BMJ.

[25] Bundesministerium für Gesundheit (2014). Shaping global health – taking joint action – embracing responsibility. The Federal Government’s Strategy Paper.

[26] Houpt ER, Pearson RD, Hall TL (2007). Three Domains of Competency in Global Health Education: recommendations for all medical students. Acad Med.

[27] Joint US/Canadian Committee on Global Health Core Competencies (2009). Global health essential core competencies.

[28] Johnson O, Bailey SL, Willott C, Crocker-Buque T, Jessop V, Birch M (2012). Global health learning outcomes for medical students in the UK. Lancet.

[29] Gruner D, Pottie K, Archibald D, Allison J, Sabourin V, Belcaid I (2015). Introducing global health into the undergraduate medical school curriculum using an e-learning program: a mixed method pilot study. BMC Med Educ.

[30] Ruiz JG, Mintzer MJ, Leipzig RM (2006). The impact of e-learning in medical education. Acad Med.

[31] Nelson BD, Kasper J, Hibberd PL, Thea DM, Herlihy JM (2012). Developing a career in global health: considerations for physicians-in-training and academic mentors. J Grad Med Educ.

[32] MD/MPH - DO/MPH Guide American Medical Student Association. http://www.amsa.org/advocacy/action-committees/cph/mdmph-domph-guide/. Accessed 16 Jan 2016.

[33] Directory of MD-MPH Educational Opportunities Association of American Medical Colleges. https://students-residents.aamc.org/applying-medical-school/article/directory-md-mph-educational-opportunities/. Accessed 24 Jan 2016.

[34] Merson M, Chapman PK (2009). The dramatic expansion of university engagement in global health: implications for U.S. policy. Report of the Center for Strategic and International Students.

[35] Rowson M, Willott C, Hughes R, Maini A, Martin S, Miranda JJ (2012). Conceptualising global health: theoretical issues and their relevance for teaching. Glob Health.

[36] Pinto AD, Cole DC, Ter Kuile A, Forman L, Rouleau K, Philpott J (2014). A case study of global health at the university: implications for research and action. Glob Health Action.

